# Mental health interventions for adolescents living with HIV or affected by HIV in low- and middle-income countries: systematic review

**DOI:** 10.1192/bjo.2020.67

**Published:** 2020-09-04

**Authors:** Arvin Bhana, Melanie Amna Abas, Jane Kelly, Myrna van Pinxteren, Lynette Alice Mudekunye, Marija Pantelic

**Affiliations:** Health Systems Research Unit, South African Medical Research Council, South Africa; and Centre for Rural Health, School of Nursing and Public Health, College of Health Sciences, University of KwaZulu-Natal, South Africa; Institute of Psychiatry, Psychology and Neuroscience, King's College London, UK; Policy and Research Directorate, Department of Community Safety, Western Cape Government, South Africa; Division of Social and Behavioural Sciences, School of Public Health and Family Medicine, University of Cape Town, South Africa; Regional Psychosocial Support Initiative (REPSSI), South Africa; Department of Social Policy and Intervention, Oxford University, UK; Frontline AIDS, UK; and Department of Medical Education, Brighton and Sussex Medical School, UK

**Keywords:** Adolescents living with AIDS, mental health, HIV/AIDS, interventions, systematic review

## Abstract

**Background:**

Mental health difficulties and mental disorders are common in adolescents living with HIV or who are affected by HIV because of living in HIV-affected households in low- and middle-income (LMICs) countries, but little is known about the interventions that target these individuals and whether they are effective.

**Aims:**

This systematic review aims to address these gaps by examining what has worked and what has not worked to support the mental health of adolescents living with HIV or affected by HIV in low- and middle-income contexts (PROSPERO Number: CRD42018103269).

**Method:**

A systematic literature review of online databases from the year 2000 to 2018, using Preferred Reporting Items for Systematic Reviews and Meta-Analyses guidelines, included English-language publications of quantitative evaluations of psychosocial interventions aiming to improve mental health among adolescents living with HIV and adolescents from HIV-affected households (aged 10–24 years) in LMICs.

**Results:**

Out of 2956 articles, 16 studies from 8 LMICs met the inclusion criteria. Thirteen studies focused on adolescents affected by HIV and only three studies on adolescents living with HIV. Only five studies included were from Sub-Saharan Africa. Interventions most often used a family-strengthening approach strengthening caregiver–adolescent relationships and communication and some problem-solving in groups or individually. Five studies reported statistically significant changes in adolescent and caregiver mental health or mental well-being, five among adolescents only and two among caregivers only.

**Conclusions:**

Research on what works to improve mental health in adolescents living with HIV in LMIC is in its nascent stages. Family-based interventions and economic strengthening show promise.

## Background

Mental health disorders among adolescents constitute a significant problem worldwide, with estimates ranging from 10 to 20%.^1^ Although the prevalence rates are less well-established in low- and middle-income countries (LMICs), estimates ranging from 13% in Brazil and India to 18% in Ethiopia and 15% in South Africa^2^ indicate similarly high prevalence rates. With almost 90% of the world's children and adolescents living in LMICs, failure to address mental health problems, which often initiate in adolescence, has wide-reaching implications for other health and developmental concerns such as lower educational achievement, substance use, violence and risk-inducing reproductive and sexual health.^2^ Adding to this burden is the growing number of young people living with HIV in LMICs: of the estimated 1.8 million adolescents living with HIV, 85% live in sub-Saharan Africa.^3^ Prevalence rates of mental health disorders in LMICs are poorly established because of a dearth of studies and of the few that exist, most are cross sectional. Findings from a study in the USA indicate 53% had a psychiatric diagnosis before HIV treatment and 44% experienced ongoing depressive disorders. However, there may be higher rates of undiagnosed depression in youth who are HIV-positive.^4^

## Relationship between HIV and mental health disorders

Even though mental health disorders are prevalent in adolescents living with HIV, a recent review indicated that not much has been done to measure the impact of these disorders or the interventions that could form a healthcare response to mental health issues.^5^ The relationship between HIV and mental health disorders is bidirectional. HIV exacerbates psychological distress among young people, and young people with mental health disorders are more likely to acquire HIV.^6,7^ Not all adolescents living with HIV will have mental health difficulties,^8,9^ but a disproportionate number experience emotional and behavioural problems at higher than expected rates, including that of other high-risk groups.^4,10,11^ adolescents living with HIV who have mental health difficulties are less likely to achieve viral suppression,^12^ have lower odds of retention in care^13,14^ and have an increased risk of AIDS-related mortality^15^ than their peers and other high-risk groups.^4,10,11^

Adolescents living with HIV face a life-long illness and must confront numerous challenges, which evolve with time. Living with HIV has an impact on multiple areas of adolescent lives: they face difficulties associated with disclosure, stigma and fear of negative reactions from others, including being bullied.^16,17^ They have to cope with the shock, fear, anger, guilt and shame of having a chronic health condition and ongoing stigmatising responses, which further has an impact on their mental health.^18^

Namibian adolescents living with HIV had greater emotional, behavioural and conduct problems compared with case controls, even after controlling for sociodemographics. Notably, after controlling for orphanhood status, mental health outcomes between the two groups were no longer significant.^19^ Compared with matched controls, adolescents living with HIV in Cape Town, South Africa had significantly poorer functional competence and self-concept and higher levels of depression, anger and disruptive behaviour.^20^ Mental health symptoms were mostly associated with sociodemographic factors and stressful life events and the loss of both parents was associated with disruptive behaviour. A recent study in Uganda of adolescents living with HIV revealed that even with antiretroviral treatment, 17% had at least one neurological disorder (enuresis/encopresis, motor/verbal tics, epilepsy), which in turn was associated with early onset of sexual intercourse.^21^ These mild-to-moderate neurocognitive disorders persist over time despite being on highly active antiretroviral treatment.^22^ But despite the heightened risk of mental health difficulties among adolescents living with HIV, evidence suggests that parent–child involvement, as well as communication and social support from peers, parents and teachers, can act as a protective factor.^11^

Adolescents who are not living with HIV but come from a household where someone else is, are also at risk for a range of mental health problems including depression, anxiety and social problems. The relationship between mental health and HIV is modified by multiple family and contextual risk influences such as the role of the caregiver,^10^ levels of impoverishment, violence, substance misuse and neighbourhood disintegration. These adolescents also experience stigma by association with family members living with HIV, which can drive persistent mental health symptoms of anxiety and depression.^23^ A systematic review of psychosocial interventions focusing on mental health resilience of young people affected by HIV showed that parenting and family support were important to their mental health and psychosocial adjustment.^8^

## Aims

Despite this growing evidence, key gaps in knowledge remain. First, the totality of the evidence base of mental health interventions for adolescents living with and affected by HIV remains unknown. Second, despite an understanding of the different mechanisms by which mental health difficulties occur for adolescents living with HIV (i.e. adolescents who were HIV-positive) versus adolescents affected by HIV (i.e. adolescents living in HIV-affected households or who are orphans because the caregiver has died from an AIDS-related illness), it is unknown whether and how the evidence base of interventions for these two populations differs. Third, a specific focus on LMICs is critical because of the limited number of mental health professionals in resource-limited settings, indicating a potential need for innovative and community-based interventions. This systematic review aims to address these gaps by examining the outcomes for interventions that have worked and that have not worked to support the mental health of adolescents in low- and middle-income contexts who are living with or are affected by HIV. Only quantitative studies regardless of the study design were included.

## Method

This paper follows the Preferred Reporting Items for Systematic Reviews and Meta-Analyses (PRISMA).^24^ As the paper is a review of published findings, ethical approval for the review was unnecessary. The protocol for the systematic review was pre-published in PROSPERO in 2018 and is available at http://www.crd.york.ac.uk/PROSPERO/display_record.asp?ID=CRD42018103269.

### Review scope

The scope of this review is restricted to intervention studies measuring a wide range of mental health outcomes among adolescents living with HIV or youth affected by HIV in LMICs between 2000 and 2018 and published in English, specifically as follows.

- Outcome: any term that refers to mental health or mental illness or emotional or psychological or adjustment or psychosocial or development or distress or trauma or self-harm or self-injury or well-being or social functioning or adaptive behaviours associated with any common or severe mental disorder, including substance use or misuse or drug misuse in adolescents living with HIV or affected by HIV.
- Population: adolescents and young people aged 10–24 years, either living with HIV or affected by HIV as a function of familial HIV.
- Geographic location: LMICs as defined by the World Bank.
- Study design: any type of quantitative evaluation of an intervention study regardless of study design.

### Search strategy

The search strategy and actual searches were conducted in June to July 2018 by an information expert (A. Bullen) using electronic databases listed below. The search strategy was piloted in PubMed (which includes Medline), followed by searches in CINAHL; Psycharticles (EBSCO); Web of Knowledge Social Science and Emerging sources databases; Cochrane; SocIndex (EBSCO); Family and Society Studies Worldwide and Academic Search Complete (EBSCO) and covered the period 2000–2018.

In addition to searching online databases, additional hand searching and citation tracking was done. Grey literature searches included Global Health database, and websites of relevant conferences, including Conferences on Retroviruses and Opportunistic Infections; International AIDS Conference; International AIDS Society and AIDS Impact. In addition to reaching out to organisational contacts, researchers in adolescent mental health were contacted for any ongoing or unpublished work. The identified studies were shared with various experts locally and internationally and authors were contacted personally where studies referred to additional work (Supplementary Appendix 1, available online at https://doi.org/10.1192/bjo.2020.67).

### Inclusion and exclusion criteria

Two reviewers (Mv.P., A.B.) independently screened titles, abstracts and full texts. Discrepancies were resolved through discussion and consensus. Papers were restricted to those reporting on quantitative evaluation. Evaluations were included if they reported on interventions with mental health outcomes among adolescents and young people aged 10–24 years living with HIV or affected by HIV in LMICs (defined by the World Bank classification). Affected by HIV includes any study of adolescents in which a caregiver is identified as living with HIV or had died because of an AIDS-related illness.

Full inclusion criteria were as follows.

- Study population: adolescents aged 10–24 years living with HIV, affected by HIV (as defined earlier).
- Study design: any quantitative study design IF they report on mental health outcomes, including randomised controlled trials, quasi-experimental designs, pre-post evaluations and post evaluations.
- Outcome measures: any measure of mental health among adolescents living with or affected by HIV, specifically measures on internalising problems (anxiety, depression), externalising problems (conduct problems, attention-deficit disorders and hyperactivity), including self-harm and substance abuse and coping or adjustment (emotional and psychological well-being, self-esteem, self-concept and resilience).

Exclusion criteria were as follows.

- Study population: adolescents aged 10–24 years whose HIV status is unknown or unspecified or the adolescent is an orphan, but the cause of the caregiver's death is unknown or not specified.
- Study design: qualitative studies; any quantitative study that does not assess any intervention effects.
- Outcome measures: measures of mental health not reported on at baseline and/or at follow-up.
- Non-English language.

### Data extraction and quality appraisal

**Fig. 1:**
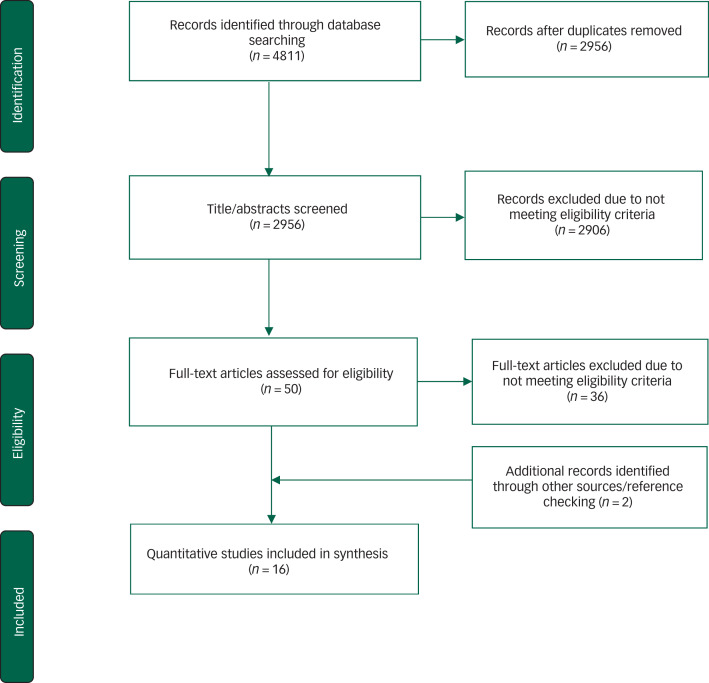
Preferred Reporting Items for Systematic Reviews and Meta-Analyses flow diagram.

Included studies were subject to detailed data abstraction and analysis. We used a predetermined data extraction tool with information about the key aspects of the study design and findings as required by PRISMA guidelines (Fig. 1). Studies were critically appraised using the Standard Quality Assessment Criteria for Evaluating Primary Research Papers from a Variety of Fields. The assessment tool included 14 criteria covering objectivity, transparency and appropriateness of research methods used (Supplementary Appendix 2).^25^

Systematic review of existing research evidence on the effectiveness and delivery of interventions to address mental health for adolescents living with HIV in low- and middle-income countries.

## Results

Of 4811 related titles, 16 mental health intervention studies were included in the review. The PRISMA flow diagram details the study exclusion process and reasons for exclusion (Fig. 1). Overall, 4133 individuals were represented in studies from China, Ethiopia, Kenya, Myanmar, Rwanda, South Africa, Uganda and Thailand.

### Study quality characteristics

Thirteen of the studies focused on HIV-affected adolescents, and three studies focused on adolescents living with HIV.^9,26,27^ Fifteen studies met 10 or more of the 14 quality criteria, most often related to inadequate participant selection details or insufficient details in the results. One study met 9 of the 14 criteria, primarily because it lacked a control group thereby increasing the chance of confounding.

#### Study design of included evaluations

Twelve studies used a randomised control trial (RCT) design.^9,26–36^ Five of these were cluster RCTs, clustered by village,^34^ township,^32^ school^33,36^ and church.^35^ One study used a quasi-experimental post-intervention design.^37^ Two studies used a pre–post design, without control groups.^38–40^

Nine studies employed a single follow-up period of either 3 or 6 months;^9,26,28,29,32,33,37–39^ six studies variously used two follow-up periods that ranged from 2, 3, 6, 9, 12 and 24 months,^27,30,34–36,40^ and one study used three follow-up periods of 6, 12 and 18 months.^31^ The median follow-up period was 3 months. Only two studies specifically focused on orphans^33,36^ and one on migrant adolescents.^38^ In the remaining studies, the orphan status of the adolescent did not feature. Given that most studies used a family-strengthening approach, it is reasonable to assume that the adolescents lived with them as a family (not necessarily as a nuclear family).

Participants were recruited through schools,^28,29,33,36,37^ healthcare settings (for example clinics or hospitals),^9,26,27,30,31,34,40^ churches,^35^ a township community sample^32^ and community-based or service delivery organisations.^38,39^

### Measurement characteristics

The majority of reviewed studies (15/16) measured adolescent mental health and/or behaviour using scales such as the Child Problem Behaviour Checklist,^29,31^ Child Depression Inventory^26,31,35–37^ and the Strengths and Difficulties Questionnaire.^9,26,32,37^ Several studies also measured caregiver mental health or stress,^9,31,39^ as well as general health or well-being of adolescents,^29,30,36^ and experiencing abuse or violence.^37,38^

Eight studies included a measure that focused on self-efficacy, self-concept or self-esteem of adolescents. For example, three studies used the Rosenberg Self-esteem Scale,^34,35,37^ while another three used the Tennessee Self-concept Scale.^9,26,36^ Three studies also measured the coping or adaptive behaviour of adolescents^9,31^ and caregivers.^31^

Several studies (11/16) incorporated measures that focused on caregiver and adolescent communication and connectedness. Scales used to assess this included Communication Comfort and Frequency^9,28,29^ and Family Connectedness.^30,40^ In addition, 11 studies also measured parenting practices using adapted measures of parenting behaviour^9,26–31,34,35,39,40^ or scales such as the Alabama Parenting Questionnaire^35^ and Parental Bonding Instrument.^34^

Seven studies measured knowledge related to HIV/AIDS using scales such as the HIV Knowledge Questionnaire,^35,36,38^ AIDS Myth Knowledge^28,29^ and the South African Demographic Health Survey.^39^ Five studies also measured stigma related to HIV/AIDS.^9,26,28,29,37^ Another four measured sexual behaviour using scales such as the Self-Efficacy to Refuse Sexual Behavior Scale^35^ and the Condom Use Self-efficacy Scale.^39^ One study measured adolescents living with HIV adherence to antiretroviral medication.^26^ Four studies looked at social support or capital using measures such as the Social Network Analysis Scale^29^ and the Network of Relationships Inventory.^35^ One study measured educational outcomes (for example, school drop-out).^36^

### Intervention characteristics

Eight studies used trained lay workers to deliver their programmes.^26,28–31,35,39,40^ Other studies used various levels of professional staff such as teachers, health educators, counsellors, social workers and mindfulness practitioners. One study used non-governmental organisation, community development, agricultural and financial officers^36^ and another used trained youth ambassadors.^37^

Intervention sites varied with five studies delivering the intervention at clinics or hospitals,^9,26,27,31,38^ four were school based,^28,29,33,37^ two studies were community based (with one delivering the intervention after weekly church services),^32,35^ two studies both community and home based^34,36^ and three were only home based.^30,39,40^ Interventions were broadly grouped into family-level and group-based interventions. The section below describes these two types of interventions.

### Family-level interventions for adolescents and caregivers

Twelve of the 16 reviewed studies focused on family-strengthening parenting interventions, all of which included both separate and joint sessions for caregivers and adolescents (Table 1).^9,26–31,34–36,39,40^

**Table 1:** Characteristics of family-based interventions studies^a^

Authors	Age group (years), setting	Study design	Participants	Intervention content	Measures	Mental health outcomes
Bhana et al^28^ (2004)	10–11, South Africa	Pilot RCT	HIV-affected adolescents Intervention *n* = 72 Control *n* = 52 Recruited through schools *n* = 124 families	10 sessions; psychoeducation: knowledge and understanding of HIV transmission; understanding developmental issues; skills development; communication; responsive parenting; monitoring/protecting adolescents; managing stigma; managing bereavement; social support; building social networks; parental and children's rights and responsibilities, stigma and dealing with bereavement	AIDS Transmission Knowledge; AIDS Myth Knowledge; parental communication styles; Parenting Communication Comfort (PCC); Parenting Communication Frequency (PCF); social support; stigma Baseline, 3 months	Only adult outcomes reported. Intervention group caregivers had greater assertive communication style, comfort in communicating difficult topics and greater social support Effect sizes:^b^ AIDS Transmission Knowledge 0.5 AIDS Myth Knowledge 0.03 Communication difficulty 0.32 Social network support 0.25
Bell et al^29^ (2008)	9–13, South Africa	RCT	HIV-affected adolescents Intervention caregivers *n* = 245 Control caregivers *n* = 233 Intervention adolescents *n* = 281 Control adolescents *n* = 298 Recruited through schools *n* = 478 families	As above	General Health Questionnaire (GHQ); Global Indicator of Well-Being Questionnaire; Revised Children's Manifest Anxiety Scale (RCMAS); AIDS Transmission Knowledge Scale; Child Problem Behavior Checklist (CBCL); Stigma Scale; Parenting Styles Scales; Family Decision-Making Questionnaire; Caregiver Monitoring Interview; PCC; PCF; Social Capital; Social Network Analysis Scales; Neighborhood Disorganization Scale; Neighborhood Social Control Scale; Neighborhood Social Cohesion Scale Baseline, 3 months	Caregiver outcomes: less stigma toward HIV-infected people; improved communication comfort and frequency; improved social support Child outcomes: less stigma toward HIV-infected people Effect size: Caregivers General health 0.30 HIV transmission knowledge 0.63 Lower external stigma 0.40 Communication comfort 0.41 Communication frequency 0.20 Adolescents AIDS Transmission Knowledge 0.50 Lower external stigma 0.70 Communication comfort 0.08 Communication frequency 0.24
Bhana, et al^26^ (2014)	10–14, South Africa	RCT	Adolescents living with HIV (ALHIV) Intervention: *n* = 33 Control: *n* = 32 *n* = 65 families	10 sessions: psychoeducation: knowledge, understanding and coping with HIV; adherence; understanding developmental issues and strategies for child safety; identity; skills development: communication; disclosure; responsive parenting; monitoring/protecting adolescents; managing stigma; managing bereavement; social support; building social networks	Youth adherence to antiretroviral treatment; Strengths and Difficulties Questionnaire (SDQ); Child Depression Inventory (CDI); Tennessee Self Concept Scale (TSCS); HIV Treatment Knowledge; Family Environment Scale (FES); Stigma Baseline, 3 months	Caregiver outcomes: less external stigma, greater communication frequency and comfort Child outcomes: none significant Effect size:^b^ CDI 0.36
Bhana et al^9^ (2016)	9–14, South Africa	RCT; pooled with pilot study	ALHIV Primary study: *n* = 111 Pilot study *n* = 66 *n* = 177 families	As above	SDQ, CDI, Beck Depression Inventory (Adults); Beck Depression Inventory II; Centre for Epidemiologic Studies Depression Scales (CES-D); FES; PCC; PCF; TSCS; Kidcope Scale Baseline, 3 and 12 months	Lower child difficulties associated with lower caregiver depression, less caregiver-reported communication about difficult issues and higher youth self-esteem Greater prosocial behaviours associated with greater caregiver-reported communication and child use of wishful thinking for coping Less youth depression associated with higher caregiver education, greater caregiver supervision and more social support seeking, higher youth self-esteem, lower internalised stigma and child use of resignation for coping Effect size:^b^ Caregivers Depression 0.50 Communication frequency 0.40 Adolescents Communication frequency 0.35 Self-concept 0.35 Coping mechanism 0.40
Betancourt et al^40^ (2014)	7–17, Rwanda	Pre–post design, no control group	HIV-affected adolescents *n* = 39 children 20 families with at least one HIV-positive caregiver	Psychoeducation about HIV and its effects on families; skills development in communication, responsive parenting, and stress management Alternatives to violence and harsh punishment; development of narrative that identifies sources of family resilience and hope from the perspective of the caregivers, the children, and a combined family narrative; problem-solving around eliciting formal and informal support structure	Family Connectedness; Good parenting; Perseverance/self-esteem; Connor-Davidson Resilience Scale (CD-RISC); Prosocial behavior; Child Discipline Household Survey (CDHS), Inventory of Socially Supportive Behaviors (ISSB); Center for Epidemiologic Studies Depression Scales for Children (CES-DC), Youth Self-Report (YSR); Irritability Questionnaire; Youth Conduct Problems Scale-Rwanda Short Form (YCPS-RS); WHO Disability Assessment Schedule for Children (WHODAS-Child) Baseline, 2 and 6 months	Caregiver-reported improvements in family connectedness, good parenting, social support and children's prosocial behavior at 2 and 6 months Improvements in caregiver-reported child perseverance/self-esteem, depression, anxiety and irritability; decreases in adolescent-reported harsh punishment and caregiver-reported harsh punishment on follow-up Effect size (6 months): Caregivers: Family connectedness 0.95 Good parenting 1.04 Self-esteem 0.85 Prosocial behavior 0.68 Social support 1.26 Depression 0.62 Anxiety 0.64 Adolescents: Irritability 0.79 Harsh punishment 0.46 Adolescent harsh punishment 0.54
Betancourt et al^30^ (2017)	7–17, Rwanda	RCT	HIV-affected adolescents Children *n* = 170, Caregiver *n* = 123, with at least one HIV-positive caregiver; *n* = 82 families	As above	Family connectedness; Good parenting; CD-RISC; Prosocial behavior, CDHS; ISSB; CES-DC; YSR; Irritability Questionnaire; YCPS-RS; WHODAS-Child Baseline, 2 and 3 months	Fewer symptoms of depression at 3 months follow-up from child self-report and caregiver reports, but no differences on conduct problems, functional impairment, family connectedness or parenting Effect size^b^ (3 months) Depression (adolescent) 0.43 Depression (caregiver report) 0.34
Eloff et al^31^ (2014)	6–10, South Africa	RCT	HIV-affected adolescents Mother–child pairs Intervention *n* = 199 Control *n* = 191; *n* = 390 families	Mother sessions: trust, living positively, disclosure, HIV and relationships, emotional experience of HIV; coping, problem-solving and stress management, human rights and stigma, parenting skills; development of children Joint sessions: getting to know each other, family memory, interaction between mother and child Mother and child sessions: planning for the future, family celebration Children: getting to know one another (2 sessions), describe self and self in family; describe self in community, identify strengths, identify coping, problem-solving, protecting self and boundaries, social skills, identifying emotions and communication skills, survival skills	CES-D, BriefCOPE; Parenting Stress Index; Coping with Children's Negative Emotions Scale; CBCL; Vinelands Adaptive Behavior Scales; CDI; RCMAS; Bar-On EQ-I – Youth version Baseline 6, 12 and 18 months	No effects on any maternal psychological or parenting measures Mothers reported improvement in child externalising behaviours, communication, daily living skills Effect size:^b^ (18 months) Adolescents: Externalising behaviour 0.27 Communication 0.22 Daily living skills .26 Socialisation 0.07 Anxiety 0.08
Li et al^34^ (2014)	6–18, China	Cluster RCT	HIV-affected adolescents Intervention *n* = 38 Control *n* = 41 *n* = 79 families	Children targeted through 5 TEA Time homework assignments and 2 family activities: (1) my family kitchen – nutrition and exercise habits, (2) my family rainbow – identify colours that represent emotions of entire family; (3) my family bag – family designs and creates a bag that reflects family values; (4) my family book requires children to fill out information about their parents, including letters to parents and grandparents; (5) my dream painting – allows children to showcase creative artwork at a community event Two family activities: (1) table topics – 100 relevant topics on index cards on dining table – children take a card and start discussions of each topic among family members; (2) family album – each family provided a digital camera to record daily life and TEA Time activity	Parental Bonding Instrument; Rosenberg Self-Esteem Scale (RSES); Problem behaviour – count of behaviours related to withdrawal, aggression, delinquency; Zung Self-Rating Depression Scale; Family Functioning Scale (only measured family cohesion, conflict and sociability) Baseline, 3 and 6 months	Increase in self-esteem for intervention children (6–12) and adolescents (13–18); improved parental care for children at 3 and 6 months; but no differences among adolescents; reduction in problem behavior (withdrawal, aggression, delinquency) among adolescents at 3 months Decline in problem behavior among children in control condition Effect size:^b^ (6 months) Parental care 6–12 years 0.36; other effect sizes could not be determined
Nestadt et al^27^ (2019)	9–14, Thailand	RCT	ALHIV Intervention: *n* = 45 Control: *n* = 43 Perinatally infected adolescents and caregivers *n* = 88 families	Concerns about growing up with HIV, communication within families, HIV stigma, HIV treatment and adherence, coping with loss/bereavement, risk behavior and responding to peer pressure, puberty and sexual relationships, future goals, and social support	SDQ; CDI; CES-D; FES, PCC; PCF; Internal/ External Stigma; FES; social support Baseline, 6 and 9 months	Significant improvements at 6 and 9 months in youth mental health and adherence, youth-caregiver communication, internalised stigma, HIV-related social support Effect size: could not be determined
Puffer et al^35^ (2016)	10–16, Kenya	Stepped-wedge cluster RCT	HIV-affected adolescents 237 adolescents, 203 caregivers *n* = 124 families	Incorporates behavioural family communication skills training, skills-based HIV prevention, behavioural parent training, cognitive–behavioural therapies Divided into 3 modules: economic empowerment – local resources, family budgeting, planning for the future; Emotional support – loving and supportive family, families that can cope with stress together; HIV education and prevention – learn about HIV together, talks about HIV and sexuality, learn about safe and healthy sexuality together	Parent-Adolescent Communication Scale; Frequency and Quality of Communication about Sex and HIV; HIV Knowledge Questionnaire; Sex Self-Efficacy; Sex Beliefs; Alabama Parenting Questionnaire, Network of Relationship Inventory; RSES; RCMAS; Multi-Dimensional Anxiety Scale for Children; CDI; SDQ Baseline, 1 and 3 months	Improved family communication that was more pronounced among youth; parenting and social support was not significant; positive effects on youth HIV knowledge and sex self-efficacy at 1, but not at 3, months No effect on youth mental health
Ssewamala et al^36^ (2016)	12–16, Uganda	Cluster RCT design	HIV-affected adolescents *n* = 1410 adolescent orphans	All groups receive counselling (priests), food, scholastic materials (1) Focus on financial literacy and asset building, future planning, family microenterprise development, and protection from risks; (2) mentorship to reinforce learning and building optimism; (3) child savings account	Primary leaving examinations score; Confidence in Achieving Education Plans; Beck Hopelessness Scale; TSCS Baseline, 24 months	Adolescent self-rated physical health increased; depression and levels of hopelessness declined Self-concept and self-efficacy improved Effect size^b^ (24 months): Hopelessness 0.43 Tennessee Self Concept 0.26
Thurman et al^39^ (2018)	12–17, South Africa	Pilot Pre–Post design, no control group	HIV-affected adolescents *n* = 105 adolescents *n* = 95 caregivers *n* = 95 families	Caregiver sessions: building a healthy family, emotional awareness, coping with sadness, coping with anger, family problem-solving skills, raising an adolescent, effective communication about emotions; behavior management, adolescent risk-taking, communicating with adolescents about sexual health, understanding HIV, preventing and responding to crises Adolescent sessions: sexual relationships, communicating about sex, HIV and sexually transmitted infections Joint sessions: getting to know one another, developing positive family relationships, problem-solving, conflict management, future planning, looking ahead	HIV Knowledge about HIV Transmission, Myths, Misconceptions; Adolescent Condom Use Knowledge; Condom Use Self-Efficacy Scale; Caregiver-Adolescent Sexual Risk Communication; Inventory of Parental and Peer Attachment; Depression Anxiety Stress Scale (DASS 21) Baseline, 9 months	Both caregiver and adolescent mental health issues (depression/ anxiety) decreased, and HIV transmission knowledge increased Effect size: could not be determined

RCT, randomised controlled trial.

a. Gender of participants: male and females.

b. Own calculation.

They all described culturally adapted parenting interventions. For example, the content and delivery of five studies (CHAMP, VUKA and VUKA Thailand) used the same methodology to meet the needs of the local context^9,26–29^ as did the two Family-Strengthening Interventions (FSI-HIV)^30,40^ and an intervention delivered at local churches.^35^ The most common elements of the family-strengthening parenting interventions broadly encompassed narratives of sources of family resilience and protective influences for the child. To strengthen understanding, studies typically included psychoeducation related to HIV transmission, treatment knowledge, understanding puberty and sexual relationships, emotional experiences related to being HIV-positive, managing internalised and external stigma and adherence. To enhance protective influences, family-strengthening studies focused on developing more responsive (less authoritarian) parenting skills, emphasising supervision and monitoring and enhancing the frequency and quality of communication skills with children and adolescents. In addition, some family-strengthening studies included coping, problem-solving (including cognitive–behavioural therapy in some cases) and stress management skills for caregivers. All these studies used trained lay health workers or counsellors to deliver these interventions.^9,26–28,31,34,39^ One study used multiple types of churches to enrol adolescents and their caregivers to participate in nine sessions of 2 h each to improve family relationships, communication skills, skills-based HIV prevention interventions, parent training and cognitive–behavioural therapies.^35^ One family-strengthening study focused specifically on economic empowerment seeking to enhance financial literacy skills, family microenterprise skills development and financial management skills^36^ and used both lay and professional staff. In this intervention, because non-governmental organisations use mentorship as part of standard care for orphaned children, an average of one mentorship meeting per month was introduced for all participants. Those in the intervention arm were offered an opportunity to open a bank account and any savings up to $10 a month per family would be matched by the project in addition to receiving ten 1- to 2-hour microenterprise development workshops (see Appendix).

### Group-based interventions for adolescents

Three studies delivered an intervention that was group-based with some taking place in schools^33,37^ or some form of group activity (Table 2).^32^ For example, school-based peer groups were used to deliver psychoeducation about fears and worries and skills development among orphans who lost one or both parents to manage their fears and increase self-esteem using individual and group problem-solving approaches to reinforce coping skills and improve self-esteem.^33^ In another example, mindfulness training and practice in the form of individual and group sessions in meditation to alter emotional and cognitive processes formed the basis of developing skills around emotional awareness, self-regulation, developing non-judgmental attitudes, listening skills and empathy and was combined with family strengthening in the form of parent training skills to enhance positive parenting skills among caregivers in a community-based study.^32^

**Table 2:** Characteristics of group-based interventions studies^a^

Authors	Age group (years), setting	Study design	Participants	Intervention Content	Measures	Mental health outcomes	Assessments
Kumakech et al^33^ (2009)	10–15, Uganda	Cluster RCT	HIV-affected adolescents *n* = 159 control *n* = 167 adolescents orphaned by AIDS	Introduction and sharing of fears, worries and concerns about orphanhood, problem-solving; HIV/AIDS; fears about orphanhood and how to handle fear, basic human needs, sources of satisfaction (to raise self-esteem) 7 key elements for each topic: Presentation of topic in encoded form such as pictures, role-play, poems, stories, or games that clearly pose the problem; identifying underlying problem; whether same problem experienced at home/school/community; reasons why problem exists; effect of the problem on them as individuals, groups and families; how they feel about the problem; what they could do as individuals, group to solve the problem	Beck Youth Inventories	Intervention had a significant impact on symptoms of anxiety, depression and anger (lower), but not self-concept among adolescents Effect size:^b^ (10 weeks) Anxiety 0.35 Depressio*n* 0.47 Anger 0.48	Baseline, 10 weeks
Mon et al^32^ (2016)	10–16, Myanmar	Cluster RCT	HIV-affected adolescents Intervention *n* = 80; Control *n* = 80;	General principles of mindfulness meditation and practice; focus on breathing to avoid intrusive thoughts and feelings; 10 min of meditation in first session and increased to 15 min in subsequent sessions Requested to practice 3–4 times a week and make notes about experience Rewarded those who practised daily with small gifts Parents/guardians requested to encourage adolescents to practice at home	Strengths and Difficulties Questionnaire (SDQ)	Emotional and conduct behaviours improved significantly at 6 months, but not social behaviours (SDQ) Effect size:(6 months) Emotional behaviour 1.8 Conduct behaviour 0.89	Baseline, 6 months
Mueller et al^37^ (2011)	8–18, South Africa	Quasi-experimental cross-sectional post-intervention design	HIV-affected adolescents Intervention *n* = 177 Control *n* = 120	Art and education activities to build self-worth, self-concept, empowerment and emotional control (self-efficacy) Activities include creating ‘hero’ books about their own life journey and group HIV education activities focused on self-advocacy and empowerment	Rosenberg Self-Esteem Scale, The Self-Efficacy Questionnaire for Children, Child Depression Inventory, SDQ, Stigma Scales, Social Connection Scale, Straus Conflict Tactics Scale, Social and Health Assessment Scale	No significant differences in depression, emotional and behavioural problems and self-esteem.	Post-intervention 6 months

RCT, randomised controlled trial.

a. Gender of participants: male and females.

b. Own calculation.

### Individual interventions for adolescents

Only one study took an individual-level approach to strengthen the mental health of adolescents affected by HIV using client-centred counselling, problem-solving therapy, group and creative therapies (music, art) as the basis of interventions among adolescent migrant workers in Ethiopia as they represented highly vulnerable mobile adolescents facing sexual abuse and exploitation (Table 3).^38^

**Table 3:** Characteristics of individual-based interventions studies^a^

Authors	Age group (years), setting	Study design	Participants	Intervention content	Measures	Mental health outcomes	Assessments
Jani et al^38^ (2016)	15–18, Ethiopia	Pre–post design, no control group	HIV-affected adolescents *n* = 1576 female and *n* = 1154 male adolescent migrants	Intervention components modelled on problem-solving therapy Included individual, group and creative therapies such as music, art and drama Counsellors addressed: (1) main issues brought forth by the client; (2) possible options and solutions to address the issues and the pros and cons of each option; and (3) plan of action selected by the client to address the problem	Youth Self Report; Anxiety Scale; Demographic Health Survey;	Reduced mental health problems (including aggressive behaviours) among female adolescents, but not among males Effect size could not be determined	Baseline, 3 months

a. Gender of participants: male and females.

### Intervention effectiveness

#### Effectiveness of family-focused interventions

Although some family-strengthening studies focused only on adolescent outcomes, others described both caregiver and adolescent outcomes separately and yet others the relative influence and association of one on the other emphasising parenting. Excluding the three pilot studies,^26,28,30^ 8 of the 12 primary studies that measured mental health and behaviour outcomes among adolescents reported that their interventions had a significant impact on adolescent mental health.^9,27,30,34,36,39,40^ In one study, caregiver reports indicated improvement in child externalising behaviours.^31^ Four studies reported no significant impact on adolescent mental health outcomes.^26,28,29,35^

In five primary studies, the interventions were associated with reduced mental health problems, including depression, anxiety, irritability and feelings of hopelessness.^30,33,36,38,39^ In one study, this was the case for female adolescents, but not for males.^38^ The authors explained that this was related to a higher prevalence of mental health problems among males at baseline and that males experienced greater social dislocation with most having lived or currently living on the street. While two studies reported no significant impact on conduct or behaviour problems,^30,37^ five reported an improvement in adolescent behaviour; specifically, externalising behaviour^31,32,34,38^ and prosocial behaviour.^40^ Using simple linear regression analysis, one family-strengthening intervention of adolescents living with HIV found that less youth depression was associated with higher caregiver education, greater caregiver supervision, more social support seeking, higher youth self-esteem, lower internalised stigma, and child use of resignation for coping.^9^ Out of the three studies that measured caregiver mental health, one reported a significant decrease in mental health issues among caregivers as a result of the intervention.^39^

One study found that fewer child difficulties were associated with lower caregiver depression.^9^ Another two studies reported an improvement in self-esteem among adolescents as a result of the intervention,^34,40^ and one reported no significant difference.^37^ However, in this same study self-efficacy improved. Lower self-efficacy was also associated with orphan status, violence at home, social connection and AIDS-related stigma. In one study both self-efficacy and self-concept improved,^36^ but in another there was no significant impact on self-concept,^33^ thus indicating equivocal results when it comes to the effect of interventions on adolescents’ self-worth.

Caregiver and child communication and connectedness improved in seven of the eight studies that measured this. For instance, compared with the control groups, caregivers and adolescents in the intervention groups felt more comfortable communicating, and communicated more frequently.^9,29,39^ Better family communication was also found among church-goers.^35^ In addition, prosocial behaviours were associated with greater caregiver-reported communication.^9^ However, one study found no significant difference in family connectedness.^30^ With regards to parenting practices more generally, the results are mixed: in two studies parenting improved; specifically caregiver monitoring^29^ and disciplinary methods.^40^ However, in three studies there was no significant difference when it came to parenting,^30,31,35^ and in another, there was a significant difference for children but not for adolescents.^34^

Three out of the six studies that measured social network or support reported a significant impact on this outcome compared with control groups.^28,29,40^ Family-level economic strengthening significantly enhanced the likelihood of taking primary leaving examinations to advance to post-primary schooling as well as influencing primary leaving examinations scores. In addition, the family-level economic strengthening arm was associated with lower levels of hopelessness among younger adolescents and a higher average self-concept compared with controls.^36^

#### Effectiveness of school and group-based interventions

A school-based group peer-support intervention revealed significant improvements in symptoms of depression, anxiety and anger among 10- to 15-year-old adolescents with HIV who were orphans.^33^ Client-centred counselling combined with group and creative arts counselling showed improved knowledge of HIV transmission, uptake of HIV and sexual health services for both male and female adolescents, but a reduction in mental health problems/behaviours (social and attention problems; anxious/depressed and aggressive behaviours) occurred only among female adolescents.^38^ A monthly group mindfulness intervention among adolescents in Myanmar showed significant improvements in emotional and conduct behaviour problems but not social behaviours.^32^ A group-based intervention among HIV-affected adolescents did not show any significant differences in depression, emotional and behavioural problems or self-esteem, although this study did not have a baseline measure.^37^

#### Associated non-mental health outcomes

There was a significant improvement in HIV knowledge in six of the seven studies that measured this outcome. For one study this occurred among caregivers;^28^ in another among both adolescents and caregivers,^29^ and four among adolescents.^26,35,36,38^ In several studies, there was an improvement in safe sex practices. For example, an increase in adolescent self-efficacy regarding condom use,^39^ and sexual risk-taking reduced in two studies.^35,36^ Adherence to antiretroviral medication improved in two out of the three studies that measured this outcome,^26^ as did internalised^26^ and externalised stigma.^29^ In the client-centred study,^38^ the intervention was associated with an increased uptake of HIV and sexual health services, including HIV testing.

## Discussion

This is the first comprehensive review of interventions aiming to support the mental health of adolescents living with and affected by HIV in LMICs. We identified 16 studies, of which 12 were family focused, 3 were group based and 1 focused on the individual adolescent.

### Key findings

#### Family-strengthening interventions hold promise

Family-strengthening interventions were most often used with both adolescents living with HIV and those affected by HIV (12 studies). Eight of these studies had a significant effect on mental health outcomes. An examination of the family-strengthening interventions that had a significant effect reveals that they were primarily focused on enhancing caregiver and adolescent resilience, improving communication and parenting skills (monitoring and supervision) and increasing social support and social networks to reduce social isolation.^9,27,30,31,34,36,39^ These elements were also most consistently reported as having significantly improved following an intervention. Nevertheless, similar elements constituted a focus of the four family-based interventions that did not work. Because studies varied in terms of sample size, study design, intervention controls and measurement, it is difficult to know what may have accounted for the non-significant findings. In part, this may be because two of these studies were pilot studies focused on establishing feasibility.^26,28^ In the other two studies, mental health outcomes were deemed to be long-term outcomes secondary to family-strengthening efforts.^29,35^

Many of the identified studies focused on strengthening families through efforts to improving parent–child relationships (parenting styles), communication and increasing knowledge related to HIV and its transmission. These studies also clearly identified the importance of caregiver well-being as well as good psychosocial support in mitigating the effects of living with or being affected by HIV. An important strength of family-focused studies is the emphasis on families and adolescents living in low-resource settings. Parental monitoring and supervision were also highlighted as important positive influences (although there is inconsistency in positive findings in the studies). This was often combined with attempts to increase knowledge around HIV transmission among both caregivers and adolescents. In addition, interventions variously included developing skills related to self-esteem, self-concept, ways of coping, problem-solving, stress management, as well as elements of cognitive–behavioural therapy. Together, it was expected that these elements would help reduce internalising stigma as well as reducing prejudice towards others affected by HIV. The overarching intervention approach was to strengthen family interactions emphasising improved parent–child communication around topics related to potential adolescent risk behaviours such as sexual debut, drug and alcohol use, negative peer influences, stigma and understanding HIV itself. The central role of the family in interventions may also be related to the targeted adolescent age ranges with eight studies recruiting samples 16 years and younger, in one from 6 to 18 years, in another from 12 to 17 years and in two from 7 to 17 years. Mental health programming that is integrated with family-strengthening efforts is likely to enhance mental health outcomes.

It is difficult to identify the elements of family-strengthening programmes that mediate improved adolescent mental health. Change may have come about through the focus on enhancing communication, promoting caring and supportive relationships and on training parents and other caregivers in skills that would be more sensitive to the emotional needs of adolescents. Most trained the adolescent and their families in problem-solving, which is an evidence-based way to improve depression. Several programmes also provided sessions to enhance knowledge and understanding of sexual and reproductive health, which, within the context of fears about HIV transmission, may positively influence mental health outcomes. The ecological model of risk and resilience extends beyond individual factors to emphasising the importance of social and cultural factors in reducing sexual risk and highlighting the protective role of family and community environments for long-term benefits, including positive effects of family-based and parenting interventions, improved communication, parent–child relationships, decreased conflict, reducing internalising and externalising symptoms, including risk behaviour. Individual well-being is dependent on both individual behaviour changes as well as contextual and cultural factors in this model.

#### What might work, but only worked in a few studies?

The four studies that did not use a family-strengthening approach used very different modalities of intervention within and between them (one of which had no significant effects on mental health^37^). Client-centred psychosocial counselling was used to deliver individual, group and creative therapies such as music art and drama as part of the intervention package. Aside from a basic single counselling session, everyone was assessed on an ongoing basis to determine further individual or group counselling. Deeply rooted emotional issues would be referred for group counselling that encompassed art, music or drama therapy.^38^ The principle of ‘conscientisation’, i.e., sharing experiential knowledge, feelings, dialogue, participation and development of critical awareness and empowerment to facilitate the transformation of oppressive experiences into liberating ones formed the basis of a school-based peer-group support interventions among adolescents orphaned by AIDS. These took the form of 16 psychosocial exercises implemented by selected teachers over 10 weeks lasting 1 h each and resulted in significant improvements in depression, anger, and anxiety but not self-concept.^33^ A mindfulness intervention involving monthly group sessions for 3 consecutive months conducted by an experienced trainer led to significant emotional and conduct behaviour improvements at 6 months but not social behaviours. Each adolescent was exposed to eight sessions lasting about 2 h. The sessions content included general principles of mindfulness meditation followed by actual practice. Each session began with mindfulness meditation increasing from 10 to 15 min with logged mindfulness practice at home three to four times per week. Guardians were only involved in providing feedback about the adolescent's practice.^32^

### Knowledge gaps and recommendations for research

Although mental health was an important outcome, attempts to directly influence mental health outcomes were found only in a minority of the studies reviewed. Further, the association between HIV and specific mental disorders is relatively understudied among adolescents living with HIV and those affected by HIV. This may be because mental health tends to be conceptualised broadly for adolescents living with HIV, as part of psychosocial well-being, alongside other socioeconomic, educational, sexual health and family-related vulnerabilities. For instance, many interventions focus on sexual risk given this was perceived as very important by study participants. Measurement bias constitutes another important gap as few studies used culturally adapted measures of mental health outcomes.

The strong focus on family-based interventions meant that assessments concentrated on caregiver and adolescent communication, and connectedness and parenting practices as these are seen to mitigate risk influences.

Aside from the relatively more frequent use of measures such the Strengths and Difficulties Questionnaire and the Child Depression Inventory, measurement characteristics of the studies varied greatly and included measures of self-esteem (Tennessee Self-concept Scale; Rosenburg Self-esteem Scale), knowledge related to HIV/AIDS, stigma and sexual behaviour. Adherence to medication was a prominent feature of the few studies in which the sample of adolescents were HIV-positive.

More importantly, well-known mental health interventions for adolescents such as cognitive–behavioural therapy or problem-solving therapy did not feature prominently, and consideration should be given to how these approaches could be incorporated with the family-strengthening elements of most interventions.

It is difficult to separate the individual effects of the more individual-strengthening elements from the family-strengthening elements as these are often reported as part of the overall intervention (even though their individual effects may be reported) and no doubt also part of the intervention. To some extent, these individual elements were added to address specific issues related to the study sample to influence individual risk as well as the risk posed by negative environments. It is also not possible to determine whether family-strengthening studies worked better with adolescents living with HIV compared with those affected by HIV, in part because only three studies^9,26,27^ focused on adolescents living with HIV (two of which used the same intervention with one being a pilot). There is also a significant dearth of intervention studies targeted to orphans living or affected by HIV. Only two studies included orphans as participants with significant positive effects.^33,36^

While every study included both female and male adolescents in their study sample, there did not appear to be any specific attempts to tailor any of the interventions to any gender. It is likely that adolescent girls and boys not only experience qualitative differences in relation to their families but may also experience specific forms of vulnerability related to their gender. Examining gender-specific vulnerabilities and its relation to mental outcomes among adolescents living with HIV and those affected by HIV should form an important part of the next generation of studies in this area.

Despite an emphasis on interventions meeting the needs of adolescents living with HIV or those affected by HIV more holistically by linking these to social protection, caregiver health, levels of household employment and sources of income,^41^ for the most part, these elements did not form a primary focus of the interventions except in one study focused on economic empowerment.^36^ With the advent of antiretrovirals being made more widely available none of the studies reported on the effects of this treatment, on self-concept or self-esteem. Greater emphasis needs to be placed on understanding the bidirectional relationship between antiretroviral treatment and mental health.

What was encouraging is that just over a third of the studies employed multiple extended follow-up assessments ranging from 1 to 3 months, while others included follow-up periods of 6, 12, 18 and 24 months. While only a few studies used a follow-up period of one year or longer,^9^^,^^30^^,^^35^ the positive intervention effects on mental health outcomes in these studies is encouraging.

Public mental health services for adolescents and their families living with or affected by HIV is an important policy issue as these remain unaddressed in current health services. Access to antiretroviral therapy alone is insufficient to ensure a good quality of life. Using implementation science approaches to scale up innovative community-based interventions that can be delivered by non-specialists, given the shortage of mental health professionals in LMICs, should be a consideration among policymakers. Given overburdened healthcare services and that most family-strengthening interventions used lay health workers, integrating interventions with current testing services should be explored.

### Limitations

Despite most of the interventions being described as family-strengthening interventions, each of these interventions used a variety of methods to deliver their interventions and used a heterogeneous group of individuals varying from trained lay counsellors to trained healthcare professionals, and it is therefore not possible to determine the scalability of these interventions. Even though most studies employed randomised study designs, the variations in sample size, study sites from school to healthcare settings, churches and community-based service delivery organisations, as well as the varying measures make direct comparisons between studies difficult. Information related to the quality and fidelity of implementation, as well as costs of the implementation are lacking. The absence of any studies specifically focused on the treatment of mental disorders is a huge research gap.

### Implications

This systematic review provides a comprehensive overview of the evidence on mental health outcomes related to adolescents living with HIV and affected by HIV. The number of evidence-based interventions that address mental health for adolescents in low- and middle-income contexts who are living with HIV is negligible and in need of urgent attention given that an increasing number of adolescents are on antiretroviral treatment. Findings among both adolescents living with or affected by HIV indicates that family-strengthening interventions are favoured over school or group-based or individual interventions. Most family-strengthening initiatives focus on enhancing caregiver parenting, communication and social connectedness than targeting any specific mental health outcome. This is primarily a function of the emphasis on building resilience in vulnerable populations from low-resource contexts as a protective mechanism for adolescents. Although the heterogeneity in study design and methods of implementation and evaluation make it difficult to directly compare the interventions, what this group of studies shows is that it is possible to influence mental health outcomes of HIV-positive adolescents or adolescents affected by HIV (see Appendix). There appears to be low uptake of these interventions, even though attempts to influence policy and practice are evident in some of the interventions. Interventions targeted at mental health concerns may need to extend the focus of family-strengthening to include a broader range of life issues associated with adolescents living with HIV and not only focus on health. This may mean that such interventions must be delivered through community-based agencies sensitive to local contexts.

## Data Availability

The authors confirm that the data supporting the findings of this study are available within the article and in the Supplementary material.

## Supplementary material

For supplementary material accompanying this paper visit http://dx.doi.org/10.1192/bjo.2020.67. click here to view supplementary material

## Appendix

### Intervention types: common characteristics

**Table:**

Intervention types	Contents	Outcomes (adolescents and caregivers)
Family-strengthening (multiple family groups)	*Psychoeducation* Knowledge and understanding of HIV transmission; Understanding developmental issues. *Skills development* Communication; Responsive parenting; Stress and conflict management; Disclosure strategies; Economic empowerment. *Problem-solvin*g Monitoring/protecting adolescents; Managing stigma; Managing bereavement; Social support.	Enhanced knowledge and understanding of HIV transmission; sexual and reproductive health; puberty, identity. Prosocial behavior; resilience; depression, anxiety, self-esteem; conduct problems; self-efficacy; family connectedness; problem-solving skills; good parenting; social support; stigma, social connection, social health assessment.
Client-centred psychosocial counselling	Client-centred psychosocial counselling delivered over 3 months based on problem-solving strategies. Minimum of 1 initial individual counselling session and if necessary, individual received further individual, group and creative therapies (music, art and drama). Counsellors were trained in adolescent health and development, psychological well-being and mental health problems, factors increasing vulnerability, counselling theories, skills and processes, group counselling, creative therapies (music, art and drama), sexual health and HIV and AIDS (knowledge, risks and prevention strategies), alcohol and drug misuse and experience with violence.	Increased knowledge and uptake of HIV and sexual health services among both males and females; 50% reduction in mental health problems (anxiety, depression, attention problems) and 60% reduction in aggressive behaviour only among females. Increased HIV knowledge, knowledge of a place to test for HIV, HIV testing and use of sexual health services increased for both males and females.
Peer-group support	16 psychosocial exercises of 1 h each using trained teachers to provide social support for improved coping among adolescents who had lost one or both parents using participatory psychosocial.	Intervention impacted on reducing anxiety, depression and anger but not enhancing self-concept scores.
Mindfulness	A total of eight group intervention sessions was provided to adolescents over 3 months, with each session lasting 2 h and facilitated by an experienced mindfulness trainer and 2 investigators. Each session covered the general principles of mindfulness meditation followed by practice and homework tasks.	Lower mean emotional and conduct behavior scores in the intervention group compared with the control group. Prosocial behavior scores did not change significantly.
