# Supplementary material for: Mental health interventions for adolescents living with HIV or affected by HIV in low- and middle-income countries: systematic review
Source: BJPsych Open. 2020 Sep 4;6(5):e104. doi: 10.1192/bjo.2020.67 (PMC7488323; doi:10.1192/bjo.2020.67)
Supplement: Supplementary file 1 [file bjosup.zip › S2056472420000678sup002.docx]

**Supplementary Appendix 1**

Search Keywords/Terms

Youth Terms:

teen* OR youth* OR adolescen* OR “young people” OR “young population” OR “young adult*” OR “young person*” OR “young wom*” OR “young man” OR “young men” OR juvenile* OR “young individual*” OR “young worker*” OR schoolleaver* OR “school leaver*” OR “high school graduate*” OR “high school age*” OR “university age*” OR boy* OR girl* OR child OR children

HIV Terms:

1. “human immunodeficiency virus” OR “human immunedeficiency virus” OR “human immuno-deficiency virus” OR “human immune-deficiency virus” OR “acquired immunodeficiency syndrome” OR “acquired immunedeficiency syndrome” OR “acquired immuno-deficiency syndrome” OR “acquired immune-deficiency syndrome” OR “Anti retroviral” OR Antiretroviral OR Anti-retroviral OR PLWH OR PLWHIV OR ALHIV OR HIV OR AIDS
2. “human immun*” AND “deficiency virus”
3. “acquired immun*” AND “deficiency syndrome”

Mental Health Terms:

1. “mental health” OR “psychosocial” OR “mental capital” OR “resilience” OR “emotional health” OR “social functioning” OR “mental illness” OR “mental disability” OR “mental distress” OR depress* OR “anorexia nervosa” OR “bulimia nervosa” OR psychotic OR psychosis OR hypochondriasis OR neurasthenia OR hyperactivity or antisocial OR “anti Social” OR anti-social OR autis* OR schizophrenia OR neurosis OR neurotic OR neuroses OR Asperger OR obsessions OR “emotional adjustment” OR “psychological distress” OR “psychosocial distress” OR “emotional distress” OR “mental trauma” OR “psychological trauma” OR PTSD OR “post trauma*” OR “self harm” OR self-harm OR suicid* OR “self mutilation” OR self-mutilation OR “self injury” OR self-injury OR anxious OR “panic attack” OR OCD OR panic OR phobi* OR dysthymi* OR somatic OR “attention deficit” OR “emotional wellbeing” OR “emotional well-being” OR “emotional well being” OR “social wellbeing” OR “social well-being” OR “social well being” OR “psychological wellbeing” OR “psychological well-being” OR “psychological well being” OR “emotional development” OR “psychological development” OR “social functioning” OR “psychological functioning” OR “emotional functioning”
2. ((emotional OR psychological OR behaviour* OR personality OR conduct OR conversion OR eating OR affective OR somatoform OR somatic OR mental OR stress OR cognitive OR obsessive OR compulsive OR bipolar OR mood OR anxiety) AND (disorder* OR problem))
3. alcoholism OR addict* OR “alcohol abuse” OR “drug abuse” OR “substance abuse” OR “alcohol misuse” OR “alcohol use disorder” OR AUD

Country Terms:

Africa OR “Central Africa” OR “Latin America” OR “Caribbean” OR “West Indies” OR “Eastern Europe” OR “Soviet” OR “South America” OR “Arab” OR “Middle East” OR “Latin America” OR “Central America” OR “Afghanistan” OR “Albania” OR “Algeria” OR “Angola” OR “Antigua” OR “Barbuda” OR “Argentina” OR “Armenia” OR “Armenian” OR “Aruba” OR “Azerbaijan” OR “Bahrain” OR “Bangladesh” OR “Barbados” OR “Benin” OR “Byelarus” OR “Byelorussian” OR “Belarus” OR “Belorussian” OR “Belorussia” OR “Belize” OR “Bhutan” OR “Bolivia” OR “Bosnia” OR “Herzegovina” OR “Hercegovina” OR “Botswana” OR “Brasil” OR “Brazil” OR “Bulgaria” OR “Burkina Faso” OR “Burkina Fasso” OR “Upper Volta” OR “Burundi” OR “Urundi” OR “Cambodia” OR “Khmer Republic” OR “Kampuchea” OR “Cameroon” OR “Cameroons” OR “Cameron“ OR “Camerons” OR “Cape Verde” OR “Central African Republic” OR “Chad” OR “Chile“ OR “China” OR “Colombia“ OR “Comoros” OR “Comoro Islands“ OR “Comores” OR “Mayotte” OR “Congo” OR “Zaire” OR “Costa Rica” OR “Cote d’Ivoire” OR “Ivory Coast” OR “Croatia” OR “Cuba” OR “Cyprus” OR “Czechoslovakia” OR “Czech Republic” OR “Slovakia” OR “Slovak Republic” OR “Djibouti” OR “French Somaliland” OR “Dominica” OR “Dominican Republic” OR “East Timor” OR “East Timur” OR “Timor Leste” OR “Ecuador” OR “Egypt” OR “United Arab Republic” OR “El Salvador” OR “Eritrea” OR “Estonia” OR “Ethiopia” OR “Fiji” OR “Gabon” OR “Gabonese Republic” OR “Gambia” OR “Gaza” OR “Georgia Republic” OR “Georgian Republic” OR “Ghana” OR “Gold Coast” OR “Greece” OR “Grenada” OR “Guatemala” OR “Guinea” OR “Guam” OR “Guiana” OR “Guyana” OR “Haiti” OR “Honduras” OR “Hungary” OR “India” OR “Maldives” OR “Indonesia” OR “Iran” OR “Iraq” OR “Isle of Man” OR “Jamaica” OR “Jordan” OR “Kazakhstan” OR “Kazakh” OR “Kenya” OR “Kiribati” OR “Korea” OR “Kosovo” OR “Kyrgyzstan” OR “Kirghizia” OR “Kyrgyz Republic” OR “Kirghiz” OR “Kirgizstan” OR “Lao PDR” OR “Laos” OR “Latvia” OR “Lebanon” OR “Lesotho” OR “Basutoland” OR “Liberia” OR “Libya” OR “Lithuania” OR “Macedonia” OR “Madagascar” OR “Malagasy Republic” OR “Malaysia” OR “Malaya” OR “Malay” OR “Sabah” OR “Sarawak” OR “Malawi” OR “Nyasaland” OR “Mali” OR “Malta” OR “Marshall Islands” OR “Mauritania” OR “Mauritius” OR “Agalega Islands” OR “Mexico” OR “Micronesia” OR “Middle East” OR “Moldova” OR “Moldovia” OR “Moldovian” OR “Mongolia” OR “Montenegro” OR “Morocco” OR “Ifni” OR “Mozambique” OR “Myanmar” OR “Myanma” OR “Burma” OR “Namibia” OR “Nepal” OR “Netherlands Antilles” OR “New Caledonia” OR “Nicaragua” OR “Niger” OR “Nigeria” OR “Northern Mariana Islands” OR “Oman” OR “Muscat” OR “Pakistan” OR “Palau” OR “Palestine” OR “Panama” OR “Paraguay” OR “Peru” OR “Philippines” OR “Philipines” OR “Phillipines” OR “Phillippines” OR “Poland” OR “Portugal” OR “Puerto Rico” OR “Romania” OR “Rumania” OR “Roumania” OR “Russia” OR “Russian” OR “Rwanda” OR “Ruanda” OR “Saint Kitts” OR “St Kitts” OR “Nevis” OR “Saint Lucia” OR “St Lucia” OR “Saint Vincent” OR “St Vincent” OR “Grenadines” OR “Samoa” OR “Samoan Islands” OR “Navigator Island” OR “Navigator Islands” OR “Sao Tome” OR “Saudi Arabia” OR “Senegal” OR “Serbia” OR “Montenegro” OR “Seychelles” OR “Sierra Leone” OR “Slovenia” OR “Sri Lanka” OR “Ceylon” OR “Solomon Islands” OR “Somalia” OR “South Africa” OR “Sudan” OR “Suriname” OR “Surinam” OR “Swaziland” OR “Syria” OR “Tajikistan” OR “Tadzhikistan” OR “Tadjikistan” OR “Tadzhik” OR “Tanzania” OR “Thailand” OR “Togo” OR “Togolese Republic” OR “Tonga” OR “Trinidad” OR “Tobago” OR “Tunisia” OR “Turkey” OR “Turkmenistan” OR “Turkmen” OR “Uganda” OR “Ukraine” OR “Uruguay” OR “USSR” OR “Soviet Union” OR “Union of Soviet Socialist Republics” OR “Uzbekistan” OR “Uzbek” OR “Vanuatu” OR “New Hebrides” OR “Venezuela” OR “Vietnam” OR “Viet Nam” OR “West Bank” OR “Yemen” OR “Yugoslavia” OR “Zambia” OR “Zimbabwe” OR “Rhodesia”

OR

“emerging market*” OR “emerging countr*” OR “Low Income Countr*” OR “middle Income countr*” OR “developing countries” OR “developing nation” OR “developing nations” OR “developing world” OR “less-developed countr*” OR “less developed countr*” OR “less-developed world” OR “less-developed world” OR “lesser-developed countr*” OR “lesser developed countr*” OR “lesser-developed nation” OR “lesser developed nation*” OR “lesser developed world” OR “lesser-developed world” OR “under-developed countr*” OR “under developed countr*” OR “under-developed nation*” OR “under developed nation*” OR “under-developed world” OR “underdeveloped world” OR “under developed world” OR “underdeveloped countr*” OR “under-developed countr*” OR “Under developed countr*” OR “under developed nation*” OR “under-developed nation*” OR “underdeveloped nation*” OR “lower middle income countr*” OR “lower middle-income countr*” OR “lower middle income nation*” OR “lower middle-income nation*” OR “upper middle-income countr*” OR “upper middle income countr*” OR “upper middle-income nation*” OR “upper middle income nation*” OR “low-income countr*” OR “low income countr*” OR “low-income nation*” OR “low income nation*” OR “lower income countr*” OR “lower-income countr*” OR “lower income nation*” OR “lower-income nation*” OR “underserved country” OR “underserved countries” OR “underserved nation” OR “underserved nations” OR “underserved world” OR “under served country” OR “under served countries” OR “under served nation” OR “under served nations” OR “under served world” OR “deprived country” OR “deprived countries” OR “deprived nation” OR “deprived nations” OR “deprived world” OR “poor country” OR “poor countries” OR “poor nation” OR “poor nations” OR “poor world” OR “poorer country” OR “poorer countries” OR “poorer nation” OR “poorer nations” OR “poorer world” OR “developing economy” OR “developing economies” OR “less developed economy” OR “less developed economies” OR “lesser developed economy” OR “lesser developed economies” OR “under developed economy” OR “under developed economies” OR “underdeveloped economy” OR “underdeveloped economies” OR “middle income economy” OR “middle income economies” OR “low income economy” OR “low income economies” OR “lower income economy” OR “lower income economies” OR “third world” OR “lami country” OR “lami countries” OR “transitional country” OR “transitional countries” OR LMIC OR LMICs OR LIC OR LICs OR UMICs OR UMIC

**Supplementary Appendix 2**

*Standard Quality Assessment Criteria for Evaluating Primary Research Papers from a Variety of Fields Leanne M. Kmet, Robert C. Lee, and Linda S. Cook.*

Checklist for assessing the quality of quantitative studies

Criteria YES (2) PARTIAL (1) NO (0)

1 Question / objective sufficiently described?

2 Study design evident and appropriate?

3 Method of subject/comparison group selection or source of information/input variables described and appropriate?

4 Subject (and comparison group, if applicable) characteristics sufficiently described?

5 If interventional and random allocation was possible, was it described?

6 If interventional and blinding of investigators was possible, was it reported?

7 If interventional and blinding of subjects was possible, was it reported?

8 Outcome and (if applicable) exposure measure(s) well defined and robust to measurement / misclassification bias?

Means of assessment reported?

9 Sample size appropriate?

10 Analytic methods described/justified and appropriate?

11 Some estimate of variance is reported for the main results?

12 Controlled for confounding?

13 Results reported in sufficient detail?

14 Conclusions supported by the results?
